# Microsphere Suspension Array Assays for Detection and Differentiation of Hendra and Nipah Viruses

**DOI:** 10.1155/2013/289295

**Published:** 2013-02-06

**Authors:** Adam J. Foord, John R. White, Axel Colling, Hans G. Heine

**Affiliations:** Australian Animal Health Laboratory, CSIRO Animal, Food and Health Sciences, Geelong, VIC 3220, Australia

## Abstract

Microsphere suspension array systems enable the simultaneous fluorescent identification of multiple separate nucleotide targets in a single reaction. We have utilized commercially available oligo-tagged microspheres (Luminex MagPlex-TAG) to construct and evaluate multiplexed assays for the detection and differentiation of Hendra virus (HeV) and Nipah virus (NiV). Both these agents are bat-borne zoonotic paramyxoviruses of increasing concern for veterinary and human health. Assays were developed targeting multiple sites within the nucleoprotein (N) and phosphoprotein (P) encoding genes. The relative specificities and sensitivities of the assays were determined using reference isolates of each virus type, samples from experimentally infected horses, and archival veterinary diagnostic submissions. Results were assessed in direct comparison with an established qPCR. The microsphere array assays achieved unequivocal differentiation of HeV and NiV and the sensitivity of HeV detection was comparable to qPCR, indicating high analytical and diagnostic specificity and sensitivity.

## 1. Introduction

Bats harbour a wide range of viruses that have been implicated in spill over events into other mammalian hosts resulting in highly virulent and often fatal zoonoses. Henipa-, filo-, lyssa-, and coronaviruses are some of the most notable examples [1]. The henipaviruses HeV and NiV are bat-borne paramyxoviruses which have been responsible for severe disease outbreaks in humans, horses, and pigs [2]. HeV was first identified in Australia in 1994 as the cause of fatal infection in horses and humans [3]. The closely related NiV was subsequently identified as the causative agent of infections in pigs and humans in Malaysia in 1998-99 [4]. The fruit bat (*Pteropus* spp.) is the only known natural reservoir of these two viruses. NiV infections in humans have been identified in several countries including Malaysia, Singapore, Bangladesh, and India with mortality up to and exceeding 75% in some of these epidemics [2]. Evidence for this virus or henipa-like viruses in bat populations in other South-East Asian locations have also been provided [5–7]. Henipa-like genomic sequences have also been detected in African bats [8] and Cedar paramyxovirus (CedPV), a novel henipa-like virus, was recently isolated in Australia [9]. HeV is endemic in Australian *Pteropus* bats and can spread directly from bats to horses, causing severe disease. Human HeV infection has so far only resulted from close contact with the blood, body fluids, and tissues of infected horses. Although bats appear to be unaffected by HeV there is a high case-fatality rate in both humans and horses and spill-over events from bats to horses are occurring with increasing regularity [10, 11]. The wide range of viruses and their enormous genome sequence variation and evolution pose a challenge to the development of molecular diagnostic assays. Although next generation sequencing can identify viruses without any prior knowledge of their sequence [12], this approach is still not practical for screening larger numbers of samples in a diagnostic context. Various combinations of conventional PCR and sequencing, or qPCR, have been used for virus identification [12, 13]. Highly conserved genes and sequences within, or across, virus species in combination with degenerate PCR primer sequences have broadened the range of viruses detectable by a single PCR [14]. The nature of these generic PCR assays necessitates the use of highly degenerate primers which can lead to a reduction in sensitivity and still requires confirmation of any resulting PCR products by DNA sequencing. Nevertheless, qPCR is highly specific, sensitive, and suitable for automation and screening of large sample numbers. However, the limited multiplexing capability of qPCR, typically no more than 2-3 combined assays, requires the setup of various and frequently, constituently different qPCR reactions when screening for multiple viruses. Microsphere suspension array assays offer advantages over qPCR in the level of readily achievable multiplexing. This allows for the simultaneous screening of many targets (up to 100 markers in the Luminex system) in a single reaction and has become a valuable tool for investigation of disease syndromes. Various assay panels for nucleic acid detection have been developed for medical or veterinary applications, including respiratory viral diseases [15], gastroenteritis pathogens [16], cystic fibrosis [17], biothreat agents [18, 19], and vesicular diseases of livestock [20]. Polymerase chain reaction amplification of the regions of interest forms the first step of these assays. Proprietary polystyrene microspheres that contain dyes displaying distinct spectral characteristics form the substrate for these assays. Luminex MagPlex-TAG microspheres (Luminex Corporation) contain unique 24 nucleotide DNA “antiTAG” sequences covalently coupled to their surface. This facilitates hybridization of specifically amplified and labeled products containing complementary “TAG” sequences and allows identification by association with particular microsphere sets in a flow cytometry-based detection system. Microsphere suspension array assays can be designed as a modular system and combined into assay panels of increasing complexity to increase the range for detection of different viruses.

Here we report the development of microsphere array assays for detection and differentiation of RNA from HeV and NiV isolates and their analytical and diagnostic performance. Furthermore, we demonstrated the utility of these assays as modules for detection of HeV in Australian horses. Our intention is to include these assays in future development of multiplex assay panels for investigating infectious diseases, syndromes in livestock, and zoonoses.

## 2. Materials and Methods

### 2.1. Viruses and Diagnostic Samples

Viruses used in this study were HeV (hendra virus/Australia/horse/Hendra/1994, six HeV isolates from four of the 2011 outbreaks in Queensland and New South Wales, Australia); NiV (Nipah virus/Malaysia/1998, Nipah virus/Bangladesh/2004); Cedar paramyxovirus; nonrelated paramyxovirus (Tioman, Sendai, Menangle, Rinderpest, and J virus); other viruses (West Nile, Kunjin, Murray valley encephalitis, and Japanese encephalitis virus). All viruses were held as stocks at the Australian Animal Health Laboratory (AAHL). Diagnostic samples used in this study included blood, tissue, and swabs from horses submitted to AAHL during several outbreaks of HeV in the Australian States of Queensland and New South Wales in 2011. Further samples from time-course experimental studies on a limited number of HeV-infected horses were also used [21]. To assist with an appropriate statistical analysis of the collective data, swab samples from submissions of quarantined European horses, presumed to be negative for HeV, were also utilized. Nucleic acid was isolated from each sample using the MagMAX 96 Viral RNA Extraction Kit (Life Technologies Cat. No. AM1836-5).

### 2.2. Microsphere Suspension Array Primer Design

**Figure 1:**
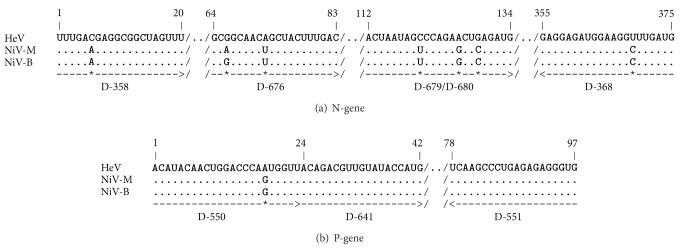
Design of oligonucleotides for henipavirus N-gene- (a) and P-gene- (b) specific microsphere array assays. Representative virus sequences are HeV (NC_001906), NiV-Malaysia (NC_002728), and NiV-Bangladesh (AY988601). Sequence gaps (sequences not displayed) outside target regions are indicated by //. Regions of sequence identity are marked by dash (-) and differences are marked (^*⋆*^) in the primer target regions. PCR primers forward (D-358) and reverse (D-368) are flanking the 375 nucleotide N-gene amplicon, and PCR primers forward (D-550) and reverse (D-551) are flanking the 97 nucleotide P-gene amplicon. Only the gene-specific target sequences and not the TAG extensions are displayed for TSPE primers (D-676, D-679, D-680, and D-641). All TSPE primers were designed to extend in forward orientation.

The primer designs (Table 1) were based on the alignments of the available 17 HeV sequences (GenBank accession numbers: HM044317, HM044318, HM044319, HM044320, HM044321, JN255800, JN255801, JN255802, JN255803, JN255804, JN255805, JN255806, JN255812, JN255814, JN255817, JN255818, NC001906) and 20 NiV sequences (GenBank accession numbers AF376747, AJ564621, AJ564622, AJ564623, AJ627196, AY029767, AY029768, AY858110, AY988601, FJ513078, FN869553, JN808857, JN808858, JN808859, JN808860, JN808861, JN808862, JN808863, JN808864, NC002728) using Geneious Pro software [23]. Primers were designed for two independent assays specific for either a 375 nucleotide region of the N-gene coding sequence (CDS) or 97 nucleotide region of the P-gene CDS of HeV and NiV (Figure 1). The N-gene assay accommodated three target-specific primer extension (TSPE) primers, one for generic detection of both HeV and NiV, and one specific for HeV or NiV only. The P-gene assay based on a qPCR assay [24] contains only a single TSPE primer for detection of both HeV and NiV. The incorporation of degenerate nucleotides was utilized to facilitate generic amplification and detection of all known HeV and NiV isolates. The design of two independent assays served as a contingency in the event of diagnostic failure should virus sequences change in an individual assay target region.

### 2.3. Microsphere Suspension Array Assay Procedure

#### 2.3.1. Primary PCR

Single-step reverse transcription PCR (RT-PCR) was performed using Superscript III One-Step RT-PCR with Platinum Taq kit (Invitrogen) with the following conditions: 25 *μ*L volume, 200 nM forward and reverse primers, and 2.0 mM MgSO_4_. Thermal cycling conditions were 30 min at 48°C (RT reaction), 2 min at 94°C (Taq activation), 45 cycles of 30 sec at 94°C, 40 sec at 50°C, and 40 sec at 68°C, followed by 68°C for 7 min. RT-PCR was performed separately for each target using the same conditions for both N and P-gene assays. The unincorporated dNTPs and primers from the initial RT-PCR were removed by treating with ExoSAP-IT (Affymetrix). Twenty-five microliters of RT-PCR were treated with 10 *μ*L ExoSAP-IT and incubated at 37°C for 30 min, followed by 10 min at 80°C to inactivate the enzymes.

#### 2.3.2. Target-Specific Primer Extension (TSPE)

Linear amplification was then performed in the presence of biotin-labeled cytosine with the required TSPE primers present in a given reaction mix. The treated RT-PCR products were then combined for TSPE reactions. The 5′ ends of the TSPE primers were designed to contain 24 base TAG sequences complementary to the particular microsphere sets, whereas the remainder of the primer sequence was designed to bind to targets within the RT-PCR product. Biotin-dCTP was incorporated in the reaction to allow detection by streptavidin-R-phycoerythrin (SA-PE). Each TSPE reaction contained 5 *μ*L of Exo-SAP-treated RT-PCR product, 0.75 U *Tsp* DNA polymerase (Invitrogen), 25 nM TSPE primer 5 *μ*M dATP/dTTP/dGTP and biotin-dCTP (Invitrogen), 1X *Tsp* DNA polymerase reaction buffer (Invitrogen), and 4.0 mM MgCl_2_. Thermocycling was performed at 95°C for 2 min, followed by 30 cycles of 94°C for 30 s, 50°C for 30 s, and 72°C for 40 s with a final extension at 72°C for 5 min.

#### 2.3.3. Microsphere Hybridisation

Products from the TPSE reactions were multiplexed with relevant MagPlex-TAG microspheres (Luminex MagPlex-TAG). Five microliters of TSPE reaction were hybridized in 50 *μ*L 1X hybridization buffer (0.2 M NaCl/0.1 M Tris/0.08% Triton X-100, pH 8.0) with 500 each (microspheres/microsphere set/well) of the appropriate MagPlex-TAG microspheres containing the antitag complimentary to the 5′ TAG on the TSPE primer. The hybridization mixture was incubated at 96°C for 90 sec and 37°C for 30 min. The microsphere mixture was transferred to a black-sided 96-well Bio-Plex flat bottom plate (Bio-Rad) and washing of magnetic microspheres was performed using an automated plate washer (Bio-Plex pro II wash station; Bio-Rad).

#### 2.3.4. Microsphere Identification and Fluorescence Detection

Seventy-five microlitres of 1X hybridization buffer containing 2 mg/L streptavidin-R-phycoerythrin (Invitrogen) were added to each plate well and the mixture was incubated in the dark at 37°C for 15 min. Instrument procedure was as described by the manufacture; briefly, 50 *μ*L of the microsphere/TSPE/streptavidin-R-phycoerythrin mixture was injected into a Bio-Plex 200 instrument (Bio-Rad), at a sample plate temperature of 37°C. Each assay plate was analysed in a Bio-Plex 200 fluorometer (Bio-Rad) running at high RP1 target setting with 100 of each microsphere set analysed per well. Fluorescence was measured as units of Median Fluorescence Intensity (MFI). A positive result was initially defined as a value greater than three times the MFI obtained from a known HeV negative control. 

### 2.4. qPCR Assays

A HeV N-gene-specific qPCR assay [24] was used for comparative assessment of the microsphere assays. Assay conditions and oligonucleotides were as described in the paper. Cut-off values were cycle threshold (CT) ≤ 40 for positive and CT ≥ 45 for negative. Results with CT values between 40 and 45 were deemed indeterminate.

### 2.5. Statistical Analysis

**Figure 2:**
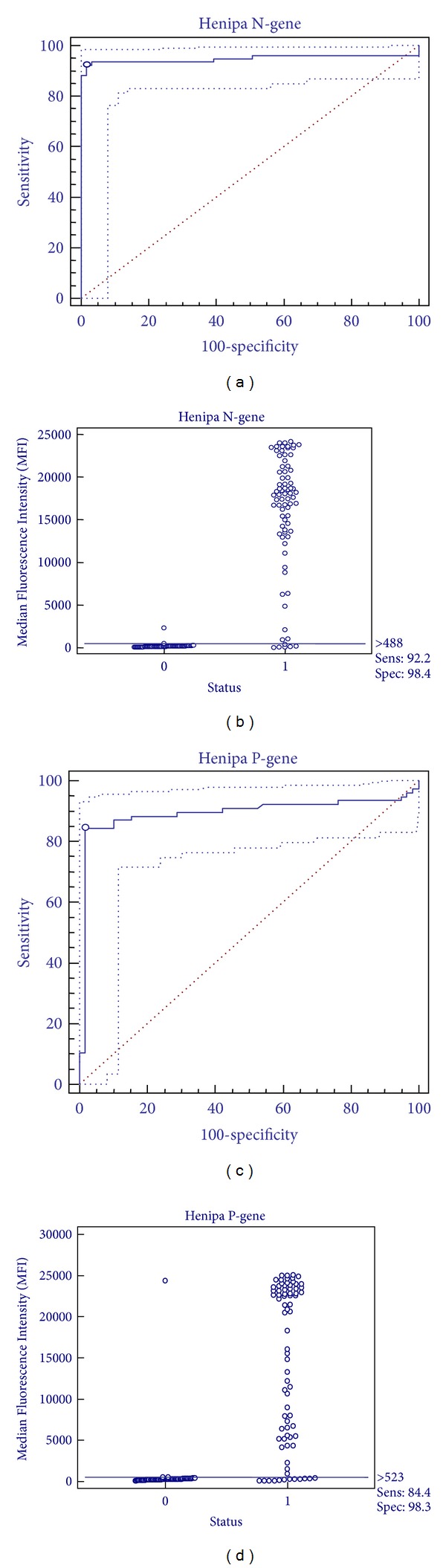
Diagnostic performance characteristics of microsphere array assays. Accuracy of microsphere array assays specific for the henipa N-gene (a, b) and henipa P-gene (c, d) was determined by receiver operating characteristic (ROC) curve analysis (a, c) using qPCR assay as reference standard. Distribution of positive (1) and negative (0) values are shown (b, d).

Statistical analyses were performed using Microsoft Excel 2007 and MedCal Version 12.3.0.0. ROC curve analysis was performed using 77 positive and 61 negative samples by qPCR for the henipa N- and P-gene microsphere array assay. For this analysis infected and noninfected horses were given the statuses 1 and 0, respectively. The area under the ROC curve (AUC) (Figure 2) was 0.948 for the henipa N (at cutoff > 488MFI) and 0.893 for the P-gene microsphere array assay (at a cut-off of > 523MFI). In the ROC analysis, values of 0.9 < AUC < 1 are considered highly accurate. A perfect test with a Se and Sp of 100% would have an AUC of 1 and be in the upper left corner of the graph [25]. An interactive dot diagram was plotted using results of the henipa N- and P-gene microsphere array assays in relation to the infected (1) and noninfected (0) category, to show false negative and false positive results at cut-offs with highest combined Se and Sp.

## 3. Results and Discussion 

### 3.1. Analytical Specificity of Microsphere Suspension Array Assays

The analytical specificity of the N- and P-gene-based microsphere array assays was assessed using RNA extracted from different virus isolates and nonrelated laboratory reference virus strains (listed in Materials and Methods). HeV and NiV isolates were positive in the generic henipavirus assays and their corresponding virus type-specific assays. All other tested viruses including CedPV were negative in the N- and P-gene assays. Although the recently isolated CedPV from Australian bats has been suggested as a henipa-like virus, it is quite distinct from HeV and NiV. Importantly, CedPV contained multiple sequence changes in each of the N- and P-gene primer regions for HeV and NiV. HeV and NiV are phylogenetically closely related having nucleotide sequence identities of 68.2% for whole genome, 78.4% for N-gene, and 70.0% for P-gene CDS, whereas the more distantly related CedPV has identities of only 47.5–48.1% for whole genome, 60.5–60.2% for N-gene, and 42.5–42.4% for P-gene CDS.

### 3.2. Analytical Sensitivity of Microsphere Suspension Array Assays

The analytical sensitivity of the microsphere array assays was assessed in direct comparison to the HeV specific qPCR assay by determining the limit of detection using tenfold serially diluted RNA template derived from HeV (Hendra virus/horse/Hendra/1994) and NiV (Nipah virus/Malaysia/1998). The microsphere suspension array assays had a dynamic range for detection equal to qPCR and were at least as sensitive as qPCR (data not shown). The fitness of microsphere array assays for the sensitive detection of HeV infection was further confirmed using archival samples from horses experimentally infected with HeV [21]. Results were compared with qPCR and loop-mediated amplification (LAMP) assays [22] (Table 2). HeV was detected by microsphere array assays as early as qPCR or LAMP assays, two days after infection. There was excellent correlation between the N- and P-gene microsphere assays. Day 2 results for horse 3 were the only occasion where an N-gene positive sample was negative in the corresponding P-gene assay. Overall, the utility of microsphere assays was confirmed by the high level of sensitivity as was apparent with positive results at days 2, 4, and 5 in horse 3 when corresponding qPCR indicated indeterminate or undetected results.

### 3.3. Diagnostic Evaluation of Microsphere Suspension Array Assays

Assays were evaluated in a retrospective analysis of archival diagnostic horse samples (*n* = 145). Expected negative values for each of the microsphere array assays were obtained from presumed negative horse samples (*n* = 40). The mean MFI (+3STD) of these samples was 121 (+120) for the henipa N-gene assay, 126 (+141) for the HeV N-gene assay, 145 (+147) for the NiV N-gene assay, and 257 (+261) for the henipa P-gene assay. Archival RNA from diagnostic submissions obtained during investigations of HeV in horses in Australia in 2011 was tested and results then correlated with the original diagnostic results obtained from qPCR assays (Table 3). Five HeV qPCR positive samples in the originally diagnostic assay were negative in both the henipa N- and P-gene assay of archival RNA (Table 3). When archival RNA was retested by qPCR all five samples were HeV negative, most likely due to degradation of the archival RNA from these samples. This indicates that the diagnostic performance of the microsphere array assays may have been underestimated in the retrospective analysis of microsphere array assays on archival RNA in comparison with original qPCR diagnostic results. Out of 7 HeV indeterminate samples (by qPCR), the henipa N-gene microsphere assay returned 4 positive and 3 negative results and the henipa P-gene 6 negative and 1 positive result. An advantage of these microsphere array assays was the clear differentiation between positive and negative results and an ability to resolve unambiguous results as exemplified by clear resolution of the indeterminate results observed at the limit of detection in qPCR. 

For ROC curve analysis (Figure 2), all of the samples positive in the N-gene henipa-specific microsphere assay were also positive in the HeV specific N-gene assay and none of these were found to be positive in the NiV-specific N-gene assay. HeV-specific positive and negative values in all assays were generally well separated, so changes from optimal cutoff values did not significantly change results in a ROC curve analysis (results not shown). For the N-gene henipa-specific assay the optimal ROC curve calculated cutoff MFI = 488 yielded sensitivity (Se) and specificity (Sp) readings of 92.2 (95% CI 83.8–97.1) and 98.4 (95% CI 91.2–100.0), respectively (Figures 2(a) and 2(b)). For the P-gene henipa-specific assay the calculated optimal cutoff MFI 523 gave an Se of 84.4 (95% CI 74.4–91.7) and an Sp of 98.3 (95% CI 90.9–100.0) (Figures 2(c) and 2(d)). The analysis of Sp and Se data for the HeV-specific assay showed near identical results to the generic henipa N-gene assay (data not shown). The ROC curve determined that negative cut-offs for all four assays were also found to exceed the highest MFI values obtained in an analysis of 40 presumed HeV negative horse swab samples in each assay. Direct comparison of all assays by ROC curve analysis using qPCR assay as reference standard identified the Henipa and HeV N-gene assays as the best performers for HeV detection. Assay accuracy for the henipa N, HeV-N and henipa P-gene assays was 0.941, 0.940, and 0.874, respectively, for samples *n* = 96 samples (qPCR confirmed), increasing to 0.948, 0.946, and 0.893 for *n* = 136 samples including 40 presumed negative samples unconfirmed by qPCR. 

The HeV N-gene test exhibited the higher Se producing less false negative results. The Sp was similar for both tests. High Se is highly desirable for a test that diagnoses zoonotic and potentially fatal disease. As the reference test (qPCR) may be imperfect, it cannot be assumed that the status of the samples is 100% accurate. Evaluation of further field samples from infected and noninfected horses would be required to obtain more robust estimates for Se and Sp.

## 4. Conclusions

The microsphere array assay modules were specific for HeV and NiV. Based on sequence conservation of target regions, these assays should detect all known HeV and NiV isolates. Furthermore, the N-gene assay reliably differentiated HeV and NiV. The analytical sensitivity of the microsphere array assays matched that of qPCR assays in the limit of detection study, the detection of virus in experimentally infected horses, and in the retrospective analysis of diagnostic field samples. The microsphere array assays are based on an open diagnostic platform allowing a high degree of customisation. This facilitates the expansion of individual assay components into larger and more complex arrays and the update of assays in response to new and emerging viruses. The microsphere array assays offer advantages over qPCR in the level of readily achievable multiplexing. Assays can be designed as a modular system and combined into assay panels of increasing complexity. This ensures that assay sensitivity and specificity are not adversely affected by difficulties often observed in multiplexed qPCR reactions. This study demonstrated the utility of the microsphere array assays for detection of HeV. Our aim is to incorporate these HeV and NiV microsphere array assays as modules in future higher multiplexed microsphere arrays. This will facilitate the development of syndrome-based assay panels for disease investigation and agent surveillance in horse, bat, pig, and human populations. 

## Figures and Tables

**Table 1:** Oligonucleotides for HeV and NiV microsphere array assays.

Name	Function	Sequence (5′-3′)	Position (^a^)
	*N-gene *		
D-358	PCR-Fwd	TTTGAMGAGGCGGCTAGTTT	125–144
D-368	PCR-Rev	CATCAARCCTTCCATCTCCTC	499–479
D-676	TSPE-henipa (*MTAG-A057)	(*)-GCRGCAACWGCTACTTTGAC	188–207
D-679	TSPE-HeV (*MTAG-A061)	(*)-ACTAATAGCCCAGAACTGAGATG	236–258
D-680	TSPE-NiV (*MTAG-A067)	(*)-ACTAATAGTCCAGAGCTCAGATG	236–258
	*P-gene *		
D-550	PCR-Fwd	ACATACAACTGGACCCARTGGTT	2698–2720
D-551	PCR-Rev	CACCCTCTCTCAGGGCTTGA	2794–2775
D-641	TSPE Henipa (*MTAG-A051)	(*)-ACAGACGTTGTATACCATG	2721–2739

(^a^) Positions are relative to the HeV genome (GenBank accession number: NC_001906). TSPE denotes target-specific primer extension.

(*) denotes sequence of 3′ extension containing anti-TAG sequence complementary to TAG sequence on microsphere as defined by particular MTAG-A# (Luminex Corporation, USA).

**Table 2:** Detection of HeV in experimentally infected horses.

Day	Microsphere array assay			
	Henipa N	Henipa P	qPCR (^a^)	LAMP (^a^)
	(MFI)	(MFI)	(Ct)	(U/Pos)
Horse 1				
0	148	213	U	U
1	185	256	U	U
2	**20 858**	**23 745**	**37.5**	U
3	**22 088**	**24 593**	**34.7**	**Pos**
4	**22 828**	**24 049**	**35.9**	**Pos**
5	**23 734**	**24 985**	**29.5**	**Pos**
6	**23 856**	**25 197**	**32.8**	**Pos**

Horse 2				
0	219	104	U	U
1	197	296	U	**Pos**
2	**22 304**	**23 547**	**36.3**	**Pos**
3	**22 983**	**23 625**	**32.4**	**Pos**
4	**11 638**	**8 142**	**38.9**	**Pos**
5	**23 063**	**24 068**	**34.3**	**Pos**
6	**22 914**	**23 333**	**31.1**	**Pos**
7	**23 024**	**24 284**	**28.1**	**Pos**
8	**22 900**	**24 145**	**29.2**	**Pos**
9	**22 900**	**24 951**	**35.2**	**Pos**

Horse 3				
0	186	301	U	U
1	221	288	U	U
2	**10 734**	375	42*	**Pos**
4	**1 271**	**2 251**	41.4*	**Pos**
5	**5 406**	**7 018**	43*	**Pos**
6	**1 804**	**8 019**	U	U
7	**16 731**	**20 575**	**37.7**	**Pos**

Pos control	23228	23451		
NTC	226	298		

Comparison of microsphere array assays performed on archival RNA extracted from daily nasal swabs of experimentally infected horses [21].

(^a^) Comparison with qPCR and loop-mediated amplification (LAMP) assay results [22] in retrospective analysis. Day indicates sampling day after challenge. MFI: median fluorescence intensity; U: undetected; Pos: positive reaction; *indicates qPCR indeterminate results; NTC: no template control. All positive results are in bold.

**Table 3:** Diagnostic evaluation of microsphere array assays for HeV detection.

Microsphere array assay (archival RNA)	HeV qPCR assay^a^		
	(original diagnostic results)		
	Positive	Negative	Indeterminate
Henipa N-gene positive	72	4	4
Henipa N-gene negative	5^b^	57	3
Total (*n* = 145)	77	61	7

Henipa P-gene positive	65	2	1
Henipa P-gene negative	12^b^	57	6
Total (*n* = 143)^c^	77	59	7

Retrospective analysis of results from microsphere array assays on archival RNA of diagnostic submissions in comparison with original qPCR diagnostic results. Preliminary cut-off values (241 MFI for the N-gene and 518 MFI for the P-gene) were derived from results of the negative horse population.

(^a^) HeV qPCR negatives include presumed HeV negative samples.

(^b^) Five samples categorised HeV qPCR positive in the originally diagnostic assay were negative in both Henipa N- and P-gene assay of archival RNA. All five samples were HeV negative when archival RNA was retested by qPCR (indicating likely degradation of the archival RNA in these samples).

(^c^) Two samples from the presumed HeV negative population were not available for the henipa P assay.

## References

[1] Calisher CH, Childs JE, Field HE (2006). Bats: important reservoir hosts of emerging viruses. *Clinical Microbiology Reviews*.

[2] Marsh GA, Wang LF (2012). Hendra and Nipah viruses: why are they so deadly?. *Current Opinion in Virology*.

[3] Murray K, Selleck P, Hooper P (1995). A morbillivirus that caused fatal disease in horses and humans. *Science*.

[4] Chua KB, Goh KJ, Wong KT (1999). Fatal encephalitis due to Nipah virus among pig-farmers in Malaysia. *The Lancet*.

[5] Wacharapluesadee S, Lumlertdacha B, Boongird K (2005). Bat Nipah virus, Thailand. *Emerging Infectious Diseases*.

[6] Reynes JM, Counor D, Ong S (2005). Nipah virus in Lyle’s flying foxes, Cambodia. *Emerging Infectious Diseases*.

[7] Sendow I, Field HE, Curran J (2006). Henipavirus in Pteropus vampyrus bats, Indonesia. *Emerging Infectious Diseases*.

[8] Hayman DTS, Suu-Ire R, Breed AC (2008). Evidence of henipavirus infection in West African fruit bats. *PLoS ONE*.

[9] Marsh GA, de Jong C, Barr JA (2012). Cedar virus: a novel henipavirus isolated from Australian bats. *PLOS Pathogens*.

[10] Smith I, Broos A, de Jong C (2011). Identifying Hendra virus diversity in pteropid bats. *PLoS ONE*.

[11] Queensland Horse Council Hendra Virus. http://www.qldhorsecouncil.com/QHC%20Documents/Notifiable%20Diseases%20Information%20Sheets/Hendra%20Virus.pdf.

[12] Drexler JF, Corman VM, Muller MA (2012). Bats host major mammalian paramyxoviruses. *Nature Communications*.

[13] Baker KS, Todd S, Marsh G (2012). Co-circulation of diverse paramyxoviruses in an urban African fruit bat population. *Journal of General Virology*.

[14] Tong S, Chern SWW, Li Y, Pallansch MA, Anderson LJ (2008). Sensitive and broadly reactive reverse transcription-PCR assays to detect novel paramyxoviruses. *Journal of Clinical Microbiology*.

[15] Jokela P, Piiparinen H, Mannonen L (2012). Performance of the Luminex xTAG respiratory viral panel fast in a clinical laboratory setting. *Journal of Virological Methods*.

[16] Liu J, Kibiki G, Maro V (2011). Multiplex reverse transcription PCR Luminex assay for detection and quantitation of viral agents of gastroenteritis. *Journal of Clinical Virology*.

[17] Johnson MA, Yoshitomi MJ, Richards CS (2007). A comparative study of five technologically diverse CFTR testing platforms. *Journal of Molecular Diagnostics*.

[18] Janse I, Bok JM, Hamidjaja RA (2012). Development and comparison of two assay formats for parallel detection of four biothreat pathogens by using suspension microarrays. *PLoS ONE*.

[19] Yang Y, Wang J, Wen H (2012). Comparison of two suspension arrays for simultaneous detection of five biothreat bacterial in powder samples. *Journal of Biomedicine and Biotechnology*.

[20] Hindson BJ, Reid SM, Baker BR (2008). Diagnostic evaluation of multiplexed reverse transcription-PCR microsphere array assay for detection of foot-and-mouth and look-alike disease viruses. *Journal of Clinical Microbiology*.

[21] Marsh GA, Haining J, Hancock TJ (2011). Experimental infection of horses with Hendra virus/Australia/horse/2008/Redlands. *Emerging Infectious Diseases*.

[22] Foord AJ, Middleton D, Heine HG (2012). Hendra virus detection using Loop-Mediated Isothermal Amplification. *Journal of Virological Methods*.

[23] Drummond AJ AB, Buxton S, Cheung M Geneious v5.4. http://www.geneious.com/.

[24] Feldman KS, Foord A, Heine HG (2009). Design and evaluation of consensus PCR assays for henipaviruses. *Journal of Virological Methods*.

[25] Greiner M, Pfeiffer D, Smith RD (2000). Principles and practical application of the receiver-operating characteristic analysis for diagnostic tests. *Preventive Veterinary Medicine*.

